# Mesenchymal vs. induced pluripotent stem cells: potential for spiral ganglion neuron regeneration in auditory neuropathy

**DOI:** 10.3389/fncel.2026.1821605

**Published:** 2026-04-29

**Authors:** Bayan Aasar, Damien Veret, Azel Zine

**Affiliations:** LBN, Univ. Montpellier, Montpellier, France

**Keywords:** auditory neuron regeneration, auditory neuropathy, induced pluripotent stem cells, mesenchymal stem cells, otic neuronal progenitors, spiral ganglion neurons, stem cell therapy

## Abstract

Auditory neuropathy is a distinct form of sensorineural hearing loss characterized by dysfunction or degeneration of primary auditory neurons, also known as spiral ganglion neurons (SGNs), which transmit acoustic information from the cochlea to the brain. Increasing evidence indicates that SGNs are particularly susceptible to degeneration induced by noise exposure, ototoxic agents, genetic mutations, or aging, often preceding the loss of cochlear mechanosensory hair cells, and thus represents a critical target for regenerative intervention for auditory neuropathy. Stem cell-based approaches have emerged as promising strategies to restore auditory nerve function. In particular, the generation of otic neuronal progenitors (ONPs) capable of replacing damaged SGNs offers a translationally relevant avenue for therapy. Among candidate sources, mesenchymal stem cells (MSCs) and induced pluripotent stem cells (iPSCs) offer distinct biological and translational advantages. iPSCs provide robust pluripotency and developmental recapitulation capacity, enabling efficient differentiation toward otic neuronal lineages, whereas MSCs offer immunomodulatory properties and paracrine neurotrophic support with lower tumorigenic risk. This mini-review critically compares MSC and iPSC-derived ONPs in terms of differentiation efficiency, neuronal maturation, integration potential, immunogenicity, and scalability. We further discuss emerging complementary strategies, including ONP transplantation, glial cell reprogramming and extracellular vesicle-based therapies. Together, these approaches highlight converging regenerative paradigms aimed at restoring auditory neuron function in neuropathic hearing loss.

## 1 Introduction

**Figure 1:**
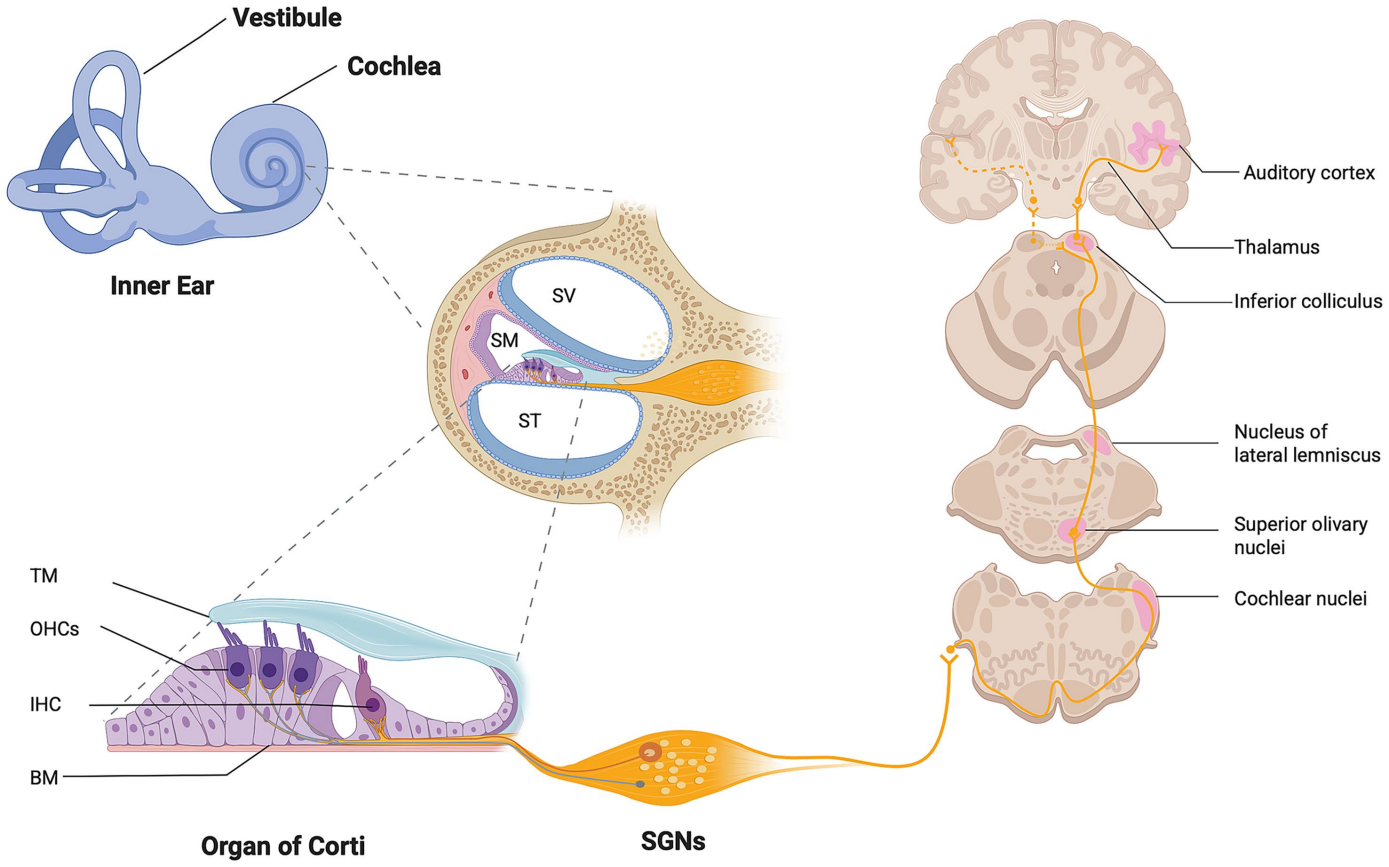
Anatomy of the cochlea and the crossed projection system to the central auditory pathway. Schematic illustration showing the anatomy of the cochlea in a mid-sagittal section. The cochlea comprises three fluid-filled chambers, separated in part by a bony structure, the osseous spiral lamina. The cochlear duct can be divided according to the type of fluid. The perilymph, contained in the Scala Vestibuli and Scala Tympani, and endolymph in the Scala Media. The SM compartment harbors the organ of Corti, where the sensory hair cells play a crucial role in converting the mechanical sound wave into an electrical neural signal that is then sent to the auditory neurons. These auditory neurons have a bipolar morphology, with a dendrite that receives information from HCs in the organ of Corti and the axon, which resides in the auditory nerve. The afferent auditory signals are then conveyed to the central auditory pathway, beginning at the cochlear nuclear complex and finalizing at the auditory cortex for sound interpretation. SV, Scala Vestibuli; ST, Scala Tympani; SM, Scala Media; IHCs, Inner hair cells; OHCs, Outer hair cells; TM, tectorial membrane; BM, basilar membrane. Created in BioRender. Zine (2026) https://BioRender.com/2si6mvf.

The mammalian inner ear contains the cochlea, responsible for sound transmission to the brain by converting auditory stimuli into neuronal impulses through a highly orchestrated mechanism. The cochlea is located in the ventral region of inner ear and is composed of three ducts: the scala vestibuli, scala media and scala tympani (Figure 1). These chambers are filled with liquids and separated by unique walls to generate fluid-based traveling waves from the base to the apex of the cochlea. The base of scala media contains the sensory epithelium, known as the organ of Corti. It comprises mechanosensory hair cells (HCs) embedded between non-sensory supporting cells and arranged in a specific mosaic pattern with HCs in luminal half and supporting cells on the basement membrane, giving rise to a pseudostratified auditory epithelium and a tonotopic axis of the cochlea. Two types of HCs exist: inner hair cells and outer hair cells, arranged in one and three rows, respectively (Figure 1). The hearing process is highly controlled by HCs and bipolar SGNs. SGNs are the primary auditory neurons responsible for transmitting synaptic input from HCs to the brain, thereby forming the neural interface between peripheral sensory transduction and central auditory processing (Figure 1) (Driver and Kelley, 2020). Among all types of hearing loss, auditory neuropathy disorder is of a particular concern. This condition, defined primarily by damage to the SGNs with relative preservation of the HCs responsible for significant hearing impairment. While the deficit of HCs can be functionally overcome by a cochlear implant, no treatment is currently available for auditory neuropathy. Reduction of otic innervation density in the inner ear does not only directly affect auditory function, but also severely limits the prospective performance of a cochlear implant (Tanna et al., 2026), highlighting the urgent need for strategies that preserve, replace, or regenerate auditory neurons. The lack of human otic neural cell bioassays forms a bottleneck in developing new biological therapies to restore lost SGNs. Among candidate stem cell sources, MSCs and iPSCs represent two biologically distinct and complementary platforms. Currently, MSCs and iPSCs can be induced in 2D and 3D *in vitro* models to generate ONPs and SGN-like cells that offer promising preclinical strategies to work toward restoring auditory neuropathy (Chen et al., 2012, 2017). In this mini-review, we compare MSC and iPSC-derived ONPs for auditory neuron regeneration in neuropathic hearing loss. We examine their biological properties, differentiation potential, safety considerations, and translational feasibility, and discuss emerging complementary strategies such as autologous stem cell transplantation, glial cell reprogramming and extracellular vesicle-based therapies. By integrating developmental, cellular, and translational perspectives, we aim to provide a conceptual framework for advancing regenerative strategies targeting auditory neuropathy.

## 2 Major causes of SGN loss

A minimum number of intact SGNs is essential to maintain normal auditory function. Therefore, the loss of SGNs leads to irreversible sensorineural hearing loss (SNHL). Ototoxic drugs, intense sound/noise, aging, or genetic factors can lead to the loss of SGNs by different mechanisms and this affect auditory function. The mature sensorineural tissues of the cochlea in mammals have very limited repair capacity and do not regenerate, so this damage is usually permanent. No clinical therapies exist thus far to rescue the dying SGNs or regenerate these cells once lost (Zhang et al., 2021).

### 2.1 Ototoxic drugs

More than 130 drugs have been found to be ototoxic, and they are divided into two groups: platinum-based antineoplastic agents and aminoglycoside antibiotics. Cisplatin is among the most used platinum-based antineoplastic drugs in clinic today to treat many types of malignant tumors. The administration of cisplatin for a short period might cause reversible damage the stria vascularis. However, when cisplatin is used for long periods and in a continuous manner, irreversible damage might occur in the cochlea, leading to SNHL. The side effects of this drug lead to apoptosis of either sensory HCs or SGNs by altering many signaling pathways that lead to cell death. On the other hand, antibiotics can lead to secondary death of SGNs due to the lack of stimulation from HCs and the lack of neurotrophic support (Steyger, 2021).

### 2.2 Noise exposure

Noise-induced hearing loss is a form of SNHL that affects more than 5% of the world population and it is more likely to be occupational (Zhang et al., 2022; Chen and Su, 2025). High intensities of noise cause temporary threshold shift that lead to the loss of ribbon synapses connecting inner HCs with SGNs. Noise can damage SGNs by several mechanisms including the neurotoxic effect of releasing excessive amounts of glutamic acid by inner HCs. This neurotoxicity is thought to be due to Ca^2+^ overload that combines calmodulin, which in turn activates several pathways related to neuron injury including mitochondria and caspase-3 mediated apoptosis. Moreover, the overactivation of glutamate receptors leads to the influx of cations and, as a result, water will passively cross the plasma membranes of SGNs which will lead to edema and death of SGNs (Zhang et al., 2022; Natarajan et al., 2024).

### 2.3 Aging

Age-related hearing loss is the number one neurodegenerative condition in aging populations. Aging damages the inner ear leading to drastic reduction of the numbers of SGNs in all regions, especially at the basal turn of the cochlea. For instance, HCs and SCs provide neurotrophins such as brain-derived neurotrophic factor (BDNF) and neurotrophic factor-3 (NTF3), essential for the survival of SGNs. However, HC death by aging can lead to later secondary death of SGNs (Elliott et al., 2022). In a study by Tang et al. (2014), they showed that the number of gamma aminobutyric acid type A receptor α1 (GABAARα1) subunits decrease with aging. GABAARs contribute to the survival of HCs and SGNs; therefore, their expression is decreased with age, leading to SNHL. Moreover, aging leads to upregulation of the expression of caspase-3 and *BAX*, and downregulation of the expression of survivor factor *BCL-2* in which intrinsic and extrinsic apoptosis pathways are activated, inducing age-related SGNs death (Frisina et al., 2016; Wang et al., 2023).

### 2.4 Genetic factors

Genetic factors are among major SNHL causes with more than 150 genes implicated, including transcription factors (i.e., *POU4F3*), ion channels (i.e., *KCNQ1, KCNE1*), extracellular matrix components, cytoskeletal proteins (i.e., Myosins), myelin proteins in Schwann cells, and even proteins of unknown functions (i.e., *DFNA5*) (Michalski and Petit, 2019). These mutations can lead to either primary or secondary degeneration of SGNs. Primary SGN degeneration normally appears with mutations in genes responsible for normal neuronal survival and regulation of synaptic transmission including *POU4F3, SLC17A8,* and *PJVK* (Ruel et al., 2008; Brooks et al., 2020; Cheng et al., 2020). Secondary SGN degeneration occurs due to mutations in genes affecting HCs and supporting cells (Bao and Ohlemiller, 2010). For instance, mutations in POU-domain and *POU4F3/pou4f3* were shown to cause hearing loss in mice and humans. Pou4f3^−/−^ mice showed degeneration of SGNs and disrupted synapses with normal HCs leading to primary SGN degeneration (Coate et al., 2012; Brooks et al., 2020). In a recent study by (Deutschmann et al. (2026), *TMPRSS3* expression was reported to contribute to the formation of excitable SGN-*like* cells in a SGN-enriched organoid model, and consequently contribute to normal SGN development in humans.

## 3 Protective and regenerative strategies for SGN loss

Substantial efforts have been made in new biological therapies to provide promising perspectives for the future of hearing restoration in SNHL. For instance, neurotrophins such BDNF and NT3 are essential for inner ear neurons survival (Elliott et al., 2021). Many molecular pathways have been implicated in protection of SGNs, including mTOR signaling, where rapamycin enhances the neurite number and length, and regulates cell death pathways (Guo et al., 2019). Other protective strategies include the modulation of the AGE-RAGE signaling pathway by soluble RAGE, combined with inhibition of apoptosis and necroptosis, and hormonal regulation, since estrogen reduces age-related apoptosis of SGNs by shifting the balance toward anti-apoptotic signaling (Wu et al., 2020). However, the inner ear is known to have physical inaccessibility due to the presence of the blood-labyrinth barrier, which forms a barrier at the luminal surface between the vasculature and fluids in the inner ear, restricting the entry of most compounds into inner ear tissue (Swan et al., 2008). Hence, there are no specific drugs approved by the FDA to prevent SGN degeneration or to regenerate new SGNs, so pharmacological therapies remain restricted in this area and scientists are currently seeking alternative methods to repair and/or regenerate new SGNs by stem cell-based therapies.

## 4 Difference between MSCs and iPSCs

**Figure 2:**
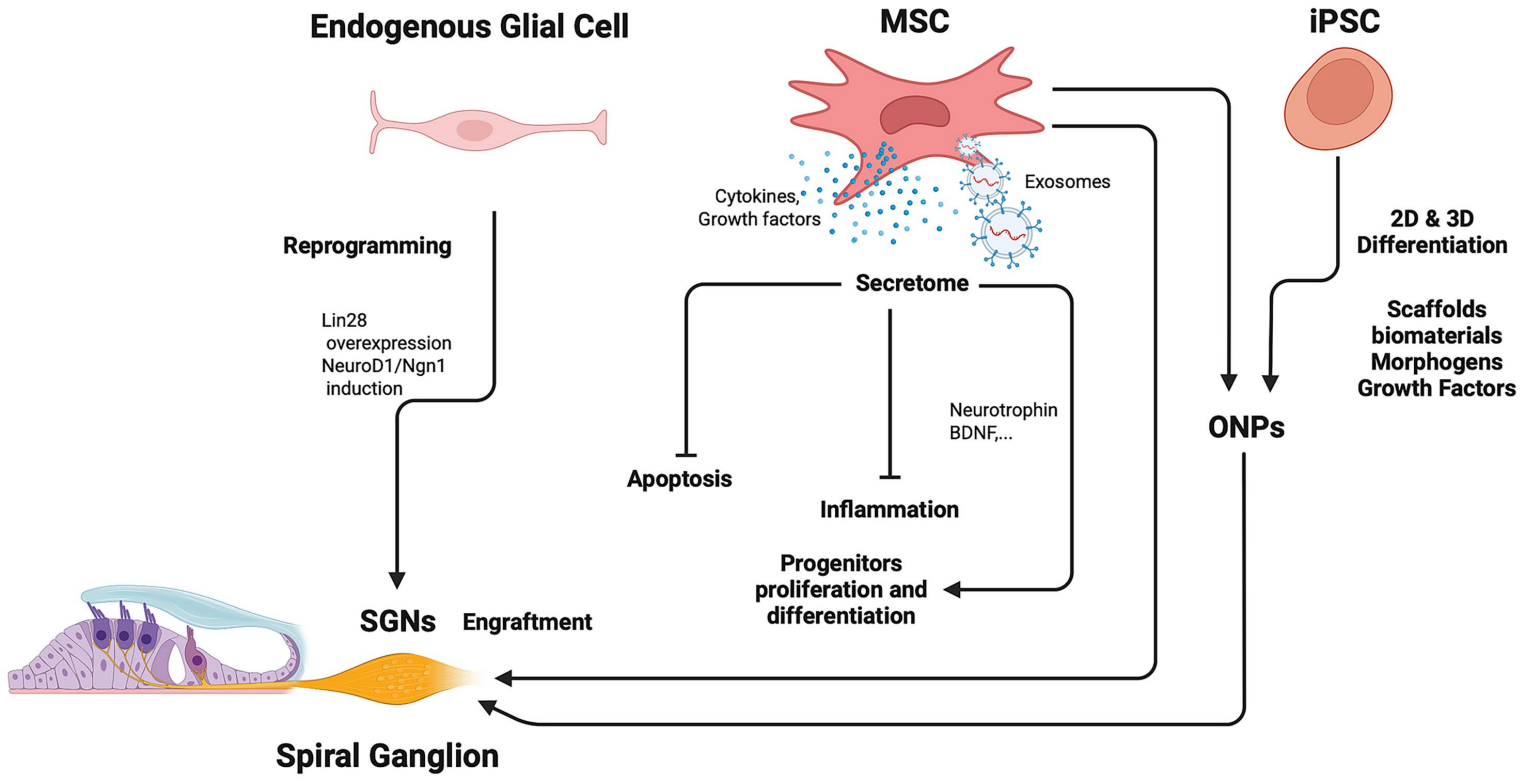
Diagram illustrating the potential of using mesenchymal stem cells (MSCs) and induced pluripotent stem cells (iPSCs) for the treatment prospects of auditory neuropathy. ONPs can be partially differentiated from MSCs and iPSCs under 2D and 3D culture systems and then transplanted into the spiral ganglion lacking or with damaged neurons. MSCs can also have a protective role to prevent SGN loss through effects of their immunomodulatory properties, rich secretome (i.e., cytokines, growth factors, neurotrophins, …) and derived exosomes. Another regenerative option is linked to the reprogramming of endogenous glial cells to SGNs that could contribute to the regeneration of SGNs and the treatment of auditory neuropathy. Created in BioRender. Zine (2026) https://BioRender.com/6y2nwfw.

In the absence of sufficient numbers of intact SGNs, none of the available current treatments are likely to be effective restoring SNHL. Therefore, it is crucial to harness the differentiation potential of stem cells as a strategy to address such hearing impairments. For instance, MSCs and iPSCs can be used to generate stem cell-derived ONPs that might be suitable as donor cells for transplantation to replace SGNs (Figure 2). During embryogenesis, the neurosensory cells of the inner ear arise from the otic vesicle (OV) (Goodrich, 2016), which is the result of invagination of the otic placode derived from non-neural ectoderm and induced by a gradient of bone morphogenetic protein (BMP) (Barth et al., 1999). Specifically, ONPs reside in the ventral region of OV and give rise to SGNs (Appler and Goodrich, 2011; Fritzsch et al., 2015). Previous studies demonstrated that the synergy between Sonic hedgehog (Shh) and retinoic acid signaling pathways promote the expression of sensory neuronal markers and support otic neuronal differentiation (Gunewardene et al., 2012). The interplay between BMP and Shh pathways, along with supplementation with BDNF and NT3, facilitates the generation of SGN-like cells from pluripotent stem cells (PSCs) (Chen et al., 2012; Matsuoka et al., 2017). By understanding *in vivo* development, researchers can recapitulate these processes using *in vitro* model systems. Human embryonic stem cells (hESCs) have been highly used in research in the last decades because they differentiate into almost all cell types. However, the ethical limitations of using embryonic cells shed light on using autologous cell types. Hence in recent years, much attention has been given to MSCs and iPSCs, especially with their accessibility, for the development of reprogramming technologies (Chen et al., 2012; Heuer et al., 2021). For successful transplantation to replace and/or repair damaged and missing cells, transplanted cells must survive, migrate to appropriate cochlear regions, and differentiate into functional neuronal phenotypes.

### 4.1 Induced pluripotent stem cells

iPSCs are adult somatic cells that have been reprogrammed to an immature, pluripotent state. This process involves four transcription factors: Oct4, Sox2, KLF4, and c-MYC oncogene (Maali et al., 2021). In the case of inner ear sensory neurons, iPSCs have been induced using *in vitro* protocols that modulate FGF, BMP, SHH and WNT pathways to drive differentiation along the otic neuronal lineage. SGN-like cells express *PAX2, PAX8, ECAD, SOX2, LMX1A/B* and *JAGGED1* at early stages and *NEUROG1, NEUROG2* and *POU4F1* at later stages (Roccio and Edge, 2019; Chizhikov et al., 2021). In a previous study (Gunewardene et al., 2014), functional otic neurons were produced from hiPSCs and characterized during their differentiation stages. The expression of PAX2, SOX2, POU4F1, and ISLET1 indicates sensory neural differentiation in parallel with the upregulation of NEUROD1, POU4F1 (Table 1). These neuron-like cells exhibited a bipolar morphology characteristic of glutamatergic neurons and were detected at 24 days *in vitro* (DIV), supporting their differentiation into otic neurons, as evidenced by the expression of vesicular glutamate transporter genes. Altogether, these findings suggest that hiPSCs can be used as invaluable sources of stem cell-derived SGNs. However, only a limited number of studies have been done to explore their neuronal otic differentiation potential and additional studies are needed in this area.

**Table 1:** Studies using mesenchymal and induced pluripotent stem cells for neuropathic hearing loss.

Cells	Source	Otic markers	Study model	Key findings	Reference
MSCs	Human bone marrow	MAP2, NF-H, GFAP	Guinea pig model of ouabain induced auditory neuropathy	Engrafted cells were detected in damaged spiral ganglion area, the number of SGNs increased with partial hearing recovery	Cho et al. (2011)
ESCs	Human ESCs	TUJ1, NKAα3, GluA2, Synaptophysin	Gerbil model of ouabain induced auditory neuropathy	Engrafted ESC-derived ONPs observed in lesioned spiral ganglion expressed Tuj1^+^ and projected fibers toward cochlear nucleus and organ of Corti. There was partial hearing recovery	Chen et al. (2012)
iPSCs	Reprogrammed human foreskin cells	BRN3A, ISLET1, NEUROD1, NEUROG1, VGLUT1	No	SGN-like cells differentiated and expressed key otic neurosensory markers *in vitro*	Gunewardene et al. (2014)
MSCs	Human nasal turbinate tissue	TUJ1	Rat cochlear explants treated with gentamicin	MSCs were engrafted into ototoxic-exposed SGN explants. Restoration of excitable SGNs was noticed in MSCs treated cultures. Some cells directly differentiated into excitable TUJ1 + neurons	Bas et al. (2014)
MSCs	Human Wharton’s Jelly	NFs, TUJ1, MAP2, CtBP2, VGLUT	No	WJ-MSCs were successfully differentiated into ONPs in culture system containing GDNF, BDNF, NT-3	Kil et al. (2016b)
MSCs	Human placenta amnion and chorion membranes	Nestin, TUJ1, NFs, MAP2, VGLUT3	Guinea pig model of neomycin induced SNHL	Engrafted MSCs improved the number of SGNs in lesioned cochlea without inflammation or immune rejection	Kil et al. (2016a)
iPSCs	Reprogrammed human dermal fibroblasts	Early otic progenitors: PAX2, PAX8, SOX2	Prenatal wildtype and CX30 mutant mice	hiPSCs were injected in prenatal mice otocyst. Engrafted cells were detected in cochlear epithelium, lateral wall and semicircular canal epithelium for up to 1 week	Takeda et al. (2018)
iPSCs	Reprogrammed human urinary cells	PAX8, PAX2, SOX2, SIX1, DLX5, EYA1, TUJ1, POU4F1, NEUROD1	Slc26a4-null mice cochlea	Transplanted OEPs migrated to organ of Corti, differentiated into HC-like cells and formed synaptic connections	Chen et al. (2018)
MSCs	Human corneal limbus (HL)	TUJ1	Mouse model of ouabain induced auditory neuropathy	Transplanted HL derived-MSCs were detected in modiolus 2 days post-engraftment. SGNs counts and ABR thresholds increased 3 months post-engraftment	Chen et al. (2019)
MSCs	Human Wharton’s Jelly	NEUROD1, BDNF, NT-3, EGF	Mouse model of noise trauma induced SNHL	MSCs were injected into the perilymph after severe noise trauma and provided significant hearing rescue. MSCs-treated animals showed preservation of some mid-turn cochlea HCs	Warnecke et al. (2021)
MSCs	Rat bone marrow	BDNF, NT-3, TUJ1, NF-200	Sprague–Dawley rat with ouabain-induced SGN degeneration	MSCs-sEV promoted SGN neurite outgrowth, rescued ouabain damaged SGNs *in vitro*, and promoted growth cone development	Chen et al. (2024)
MSCs	Human umbilical cord	MYO7A	Mouse model of neomycin induced SNHL	MSCs-derived exosomes were successfully injected in the round window and significantly reduced HC loss, increased autophagy, and reduced ABR thresholds	Liu et al. (2024)

MSCs, mesenchymal stem cells; iPSCs, induced pluripotent stem cells; ESCs, embryonic stem cells; HCs, hair cells; ONPs, otic neuronal progenitors; OEPs, otic epithelial progenitors; SGNs, spiral ganglion neurons; SNHL, sensorineural hearing loss; ABR, auditory brainstem response; EV, extracellular vesicles; BDNF, brain-derived neurotrophic factor; EGF, epidermal growth factor; GDNF, glial-derived neurotrophic factor; NT-3, neurotrophin 3.

### 4.2 Mesenchymal stem cells

Mesenchymal stem cells are non-hematopoietic adult stromal cells, characterized by their proliferative and self-renewal capacity, their adherence to plastic, and paracrine and migration activity.

The presence of MSCs in tissues is identified by the expression of the surface antigens CD105, CD90 and CD73 (Ding et al., 2011). It has been shown that several MSCs can differentiate into otic sensory neurons including olfactory/nasal MSCs (Bas et al., 2014), Wharton’s jelly-derived MSCs (Kil et al., 2016b), and bone marrow-derived MSCs (Cho et al., 2011) (Table 1). In a recent study, Messat et al. (2024) generated otic neural progenitors (ONPs) and SGN-like cells from human dental pulp stem cells by sequential treatment with SB/LDN and SB/BMP4 up to 7 DIV, followed by BDNF/NT3 until 21 DIV, using a neutrosphere-based culture system. By 13 DIV, the ONPs expressed BMP7, PAX2, SOX2, NEUROD1, and NEUROG1, indicating early commitment toward an SGN-like phenotype, concomitant with the downregulation of early otic markers. At 32 DIV, approximately 40% of cells co-expressed POU4F1 and SOX2, while additional SGN markers were detected at both 21 and 32 DIV. Furthermore, atomic force microscopy analysis demonstrated that the differentiated cells exhibited nanomechanical properties such as cellular stiffness (Young’s modulus), and elasticity comparable to those of native SGNs isolated from the postnatal rat inner ear. These parameters reflect the cytoskeletal organization and biomechanical integrity of the cells, thereby providing functional evidence that the derived SGN-like cells recapitulate key physical characteristics of their *in vivo* counterparts.

## 5 Therapeutic approaches for neuropathic hearing loss using ONPs derived from MSCs and iPSCs

Technological advances are providing new and promising tools for exploring new avenues for the treatment of hearing loss caused by SGN neurodegeneration. These strategies could include regeneration, reprograming, and/or protection from trauma induced SGN loss. The challenge in this field is to keep SGN-like cells stable and functional and to avoid unsuccessful transplantation into the inhospitable environment of the inner ear. To keep stem cells in near native surroundings, most culture conditions are done in 3D otic-like cell models.

### 5.1 Regeneration of SGNs by engraftment of MSCs and iPSCs-derived ONPs

Transplantation of PSCs and adult stem cells as donor cells has been discussed and evaluated in several reviews (Creber et al., 2025) and hence, we will briefly focus on recent developments and the few studies on engraftment of human PSCs-derived otic progenitors into the cochlea. Transplantation of partially differentiated ONPs could be safer and more efficient, and most importantly generate both HCs and neuron-like cells. Transplantation of hESC-derived ONPs into the gerbil with selective ablation of SGNs has led to partial improvement in the auditory brainstem responses 10 weeks post-engraftment. These investigators observed neuronal cell somas with processes that extend in opposite directions to both HCs and to the cochlear nucleus (Chen et al., 2012). In another study, transplantation of hiPSC-derived otic epithelial progenitors resulted in better survival and engraftment in the otocysts of Connexin30-knock-out mice compared to their engraftment into adult cochleae (Takeda et al., 2018). Furthermore, hiPSC-derived otic progenitors have been injected into the modiolus of gerbil cochleae and migration and engraftment of the transplanted otic progenitors has been reported (Chen et al., 2018). Our group has, in a previous study successfully engrafted hiPSC-derived otic progenitors into the cochlear sensory epithelium in an optimized guinea pig model of amikacin induced-ototoxicity (Lopez-Juarez et al., 2019). In this study, partially differentiated ONPs infused into the scala tympani migrated and survived up to 4 weeks post-engraftment and displayed immunophenotypes of sensory differentiation. By combining stem cells with biomaterial scaffolds in the inner ear, neuronal induction of hiPSCs in a 3D-collagen scaffold followed by transplantation into the scala tympani of the guinea-pig cochleae successfully engrafted into the auditory nerve (Ishikawa et al., 2017). This transplantation paradigm resulted in glutamate transporter (VGlut1) expression in more than 50% of the transplanted ONPs. Future studies should systematically evaluate the engraftment potential of human iPSC and MSC derived ONPs in robust models of auditory neuropathy.

### 5.2 Regeneration of SGNs by endogenous glial cell reprogramming

Glial cells in the inner ear consist of Schwann cells that distribute along the fibers of SGNs and satellite glial cells that reside in Rosenthal canal surrounding SGN cell bodies. It has been reported that glial cells in the inner ear play a vital role in protecting SGNs from degeneration and helping SGNs to perform their normal functions (Akil et al., 2015). The Schwann cells along the neuronal fibers express multiple neurotrophins, including BDNF and NT-3, that induce the development and survival of SGNs (Hansen et al., 2001). In an interesting study (McLean et al., 2016) found that glial cells in the inner ear could also become a promising resource for SGN regeneration. They demonstrated that proteolipid protein (PLP1) + glial cells derived from the newborn mouse spiral ganglion could give rise to multiple cell types *in vitro*, including glial cells and neurons, and thus the PLP1 + glial cells existing in the inner ear were identified as neural progenitors. In another study (Li et al. 2020) also found that glial cells in the inner ears of mice started to express SGN markers *in vivo* 6 days after ectopic expression of *Neurog1* and *Neurod1*. Moreover, part of the newly generated SGNs exhibited a similar cellular phenotype to that of native SGNs. In the same line of evidence, transient Lin28 overexpression in Plp1-expressing glial cells induced expression of neural stem cell markers and subsequent conversion into otic neurons (Kempfle et al., 2020) (Figure 2). This suggests the potential for inner ear glia to be converted into otic neurons as a regeneration therapy for neural replacement in auditory neuropathy. However, because of lack of an effective method for separating Schwann and satellite glial cells in the inner ear, the respective function of Schwann cells and satellite glial cells in SGN regeneration remains to be defined.

### 5.3 Protective effect of MSCs and MSC-derived exosomes in auditory neuropathy

#### 5.3.1 Effect of MSCs

As a result of their immunomodulatory activity and trophic support function, MSCs are invaluable cells that provide paracrine neuroprotective support to SGNs in SNHL. A central strategy to enhance neuronal differentiation and SGN preservation involves the use of neurotrophins (Zine and Fritzsch, 2023; Pavlinkova et al., 2026). These neurotrophins are supplied either in neuronal medium for the complete differentiation of MSCs to neuronal cells *in vitro* (Chorath et al., 2020) or as genetically modified MSCs in a guinea pig model of cochlear implantation. Notably, BDNF-producing MSCs encapsulated within alginate-based matrices have been shown to preserve SGN survival and limit degeneration. Moreover, MSCs derived from Wharton’s jelly have been induced *in vitro* toward auditory neuronal-like cells by a neurotrophic stimulation, suggesting a favorable stem cell source for regenerative applications (Kil et al., 2016b).

#### 5.3.2 Effect of MSC-derived exosomes

MSCs-derived exosomes are a cell-free therapeutic strategy characterized by extracellular vehicles (EVs), similar to the paracrine and signaling activity of MSCs including miRNA, mRNA, and proteins. Therefore, they can be used in SNHL treatment with minimal safety and immunoreaction concerns. Although a limited number of studies exists addressing the potential of MSC-derived exosomes in SNHL, preclinical findings are promising. In one study, hUC-MSC-exosome enhanced survival and neurite growth in rats, in which hearing loss was partially restored. Gene panel analysis revealed that exosomes can modulate the expression of certain genes related to neuroprotective and tissue repair properties. Exosomes derived from inner ear tissues have also shown otoprotective effects (Tsai et al., 2021). For instance, exosomes from inner ear stem cells prevented gentamicin-induced ototoxicity (Lai et al., 2020), while those from cochlear spiral ganglion progenitors inhibited inflammation and attenuated ischemia–reperfusion-induced cochlea damage (Yang et al., 2021). While MSC-derived exosomes are thought to be effective in reducing SNHL symptoms, no clinical trials have been performed to evaluate them in SNHL patients due to some limitations including variability of MSC sources, isolation techniques and administration routes.

## 6 Conclusion and future perspectives

Regeneration of SGNs represents a central challenge in the treatment of auditory neuropathy. Both MSCs and iPSCs provide promising yet distinct biological frameworks for therapeutic intervention. iPSCs exhibit strong potential for neuronal replacement through developmental recapitulation and lineage specification. In contrast, MSCs offer robust neuroprotective, immunomodulatory, and paracrine effects with lower safety concerns. Despite encouraging preclinical findings, critical barriers persist, such as achieving long-term survival and functional integration of transplanted ONPs, and precise axonal connectivity with HCs and the brain. In addition is the need to develop scalable, GMP-compliant manufacturing pipelines with safety controls. Future research should prioritize combinatorial regenerative strategies, advanced biomaterial-guided delivery systems, and *in situ* reprogramming approaches. A deeper understanding of the auditory nerve microenvironment will be essential to translate stem cell-based therapies from experimental models to auditory neuropathy treatments.
